# Circulating ceramide levels and ratios in Emirati youth under 18 years: associations with cardiometabolic risk factors

**DOI:** 10.1186/s12944-024-02080-6

**Published:** 2024-04-01

**Authors:** Youssef M. Shalaby, Bashar Al-Zohily, Anjana Raj, Javed Yasin, Sania Al Hamad, Charalambos Antoniades, Nadia Akawi, Elhadi H. Aburawi

**Affiliations:** 1 Department of Genetics and Genomics, College of Medicine and Health Sciences, United Arab Emirates University, Al-Ain, United Arab Emirates; 2 Department of Internal Medicine, College of Medicine and Health Sciences, United Arab Emirates University, Al-Ain, United Arab Emirates; 3 Department of Paediatrics, College of Medicine and Health Sciences, United Arab Emirates University, Al-Ain, United Arab Emirates; 4 Division of Cardiovascular Medicine, University of Oxford, Oxford, UK; 5 Department of Pharmacology and Toxicology, Faculty of Pharmacy, Ahram Canadian University, 6th of October City, Egypt

**Keywords:** Ceramide, Reference intervals, Risk score, Cardiometabolic diseases

## Abstract

**Background:**

Circulating ceramide (Cer) drives various pathological processes associated with cardiovascular diseases, liver illness, and diabetes mellitus. Although recognized as predictors of cardiometabolic diseases (CMD) in research and clinical settings, their potential for predicting CMD risk in individuals under 18 remains unexplored.

**Objectives:**

This study was designed to utilize Liquid Chromatography-Mass Spectrometry (LC-MS/MS) methodology to determine the biological reference ranges for Cer in plasma samples of Emirati children and develop a risk assessment score (CERT-1) based on Cer concentrations.

**Methods:**

Using LC-MS/MS, we developed a method to measure five Cer species in plasma samples of 582 Emirati participants aged 5–17. We used the circulating concentrations of these Cer to determine their reference intervals in this population. We employed traditional statistical analyses to develop a risk score (CERT-1) and assess the association between Cer levels and conventional biomarkers of CMD.

**Results:**

We validated a high-throughput methodology using LC–MS/MS to quantify five Cer species in human plasma. Reference values for this population (*n* = 582) were quantified: CerC16:0 (0.12–0.29 µmol/L), CerC18:0 (0.019–0.067 µmol/L), CerC22:0 (0.102–0.525 µmol/L), CerC24:0 (0.65–1.54 µmol/L) and CerC24:1 (0.212–0.945 µmol/L). We devised a risk assessment score (CERT-1) based on plasma Cer content in the study participants, showing that 72.5% have low to moderate risk and 9.3% are at a higher risk of developing CMD. Our analyses also revealed a significant correlation (*P* < 0.05) between this score and the conventional risk factors linked to CMD, indicating its potential clinical implication.

**Conclusion:**

This study presents a clinical-scaled LC–MS/MS methodology for assessing clinically relevant Cer, setting reference ranges, and developing a risk score (CERT-1) for young Emirati individuals. Our findings can enhance primary risk prediction and inform the management and follow-up of CMD from an early age.

**Supplementary Information:**

The online version contains supplementary material available at 10.1186/s12944-024-02080-6.

## Introduction

The prevalence of childhood obesity is rising globally, specifically in the UAE, where it has been reported that over 2% of children become obese each year in a population-based study utilizing student anthropometric measurements [1]. This affects the lives of many and raises the issue of potential long-term health outcomes, such as an increased risk of cardiometabolic disorders (CMD) development in the future. This problem can be tackled proactively to prevent health and economic burdens on society [1]. Even though traditional risk factors of CMDs in adults are well known, hardly any research took a closer look at the possible role of early markers of CMD, such as ceramides (Cer), in foreseeing the future risk, especially in a relatively young population.

Cer are a class of lipids that have attracted substantial interest in molecular biology and medicine due to their multifaceted roles in various physiological processes and disease mechanisms [2, 3]. These proapoptotic lipids, composed of sphingosine and fatty acid, have been implicated in numerous cellular pathways and functions, often with detrimental consequences. Cer have been involved in various CMDs due to their capacity to disrupt insulin signaling pathways, impede endothelial function, and activate inflammatory signaling pathways [3]. Emerging evidence implicates Cer in liver disease as it was found an increase in Cer production is associated with the development of non-alcoholic fatty liver disease in humans. As a result, lipotoxicity occurs when these Cer bring deleterious effects on hepatic metabolism. This eventually leads to fibrosis and cirrhosis [4].

Furthermore, the liver can assess intracellular levels of Cer and respond to their overproduction by enhancing the secretion of Cer, mainly within very low-density lipoprotein, into the blood circulation [5]. Therefore, the liver is one of the significant sources of circulating Cer. Currently, systemic or plasma ratios of Cer species, particularly CerC16:0, CerC18:0, and CerC24:1, are used as powerful predictors for the onset of type 2 diabetes and adverse cardiovascular outcomes [6–8]. Increased concentrations of these Cer species in the bloodstream have been linked to a heightened risk of severe complications such as myocardial infarction, acute coronary syndromes, and mortality. A stronger association was observed for the ratios of these Cer to CerC22:0 and CerC24:0 with cardiovascular adverse events and death [9–11]. Consequently, risk scores such as the CERT-1 score, formulated based on measuring these circulating Cer and their ratios, have been integrated into regular clinical practice [12].

A collaborative initiative is underway to standardize the methods for quantifying targeted Cer across mass spectrometry-based platforms and among various laboratories globally [13]. The primary objective of the Cer Ring Trial is to establish the upper and lower concentration boundaries of Cer, allowing for an assessment of the physiological and pathological biological variations of these crucial lipids across different populations. Notably, the Middle East, including the United Arab Emirates (UAE), is not currently participating in this trial, and there is no published data concerning the normal levels of circulating Cer in this region. Consequently, we have developed and validated a methodology using liquid chromatography-tandem mass spectrometry (LC-MS/MS) suitable for large-scale clinical measurements of the five distinct Cer previously implicated in CMD risk. This study was applied to precisely determine the concentrations of Cer in a cohort of young individuals from the UAE. Furthermore, it was used to construct the CERT-1 score, allowing for the early assessment of CMD risk.

## Materials and methods

### Study population and parameters

The study population consisted of children aged 5–17 during the examination. Abu Dhabi Education Council (ADEC) office was approached to get their official permission to run this cross-sectional survey in Al Ain city national schools. We have employed a two-stage sampling technique to select the schools and the students within each school. The schools in UAE are sex-specific, so we stratified them to collect a similar number of girls and boys. Out of 170 schools in Al Ain, 34 schools were randomly selected by using a random number table as the first stage sampling frame based on school size and the percentage of boys and girls registered in each school. Furthermore, the students were randomly selected through a systematic sampling procedure via a computer-generated list of random numbers in this second sampling stage by selecting even numbers of students according to the ADEC school.

The study protocol was approved on 4 June 2019 by the Human Ethics Committee, College of Medicine and Health Sciences, United Arab Emirates University (AAMD-ERH_2019), Al Ain, UAE.

The aims, scope, and importance of the study were explained to children’s parents, and written consent was obtained. Participants’ personal information was anonymized, and confidentiality was maintained. Investigations were conducted according to the principles outlined in the Declaration of Helsinki and European guidelines for clinical research. One of the child’s parents completed an informed written consent form before sample collection. The enrollment process involved 582 young people 5–17 years of age, and the demographics, along with laboratory parameters of the non-fasting blood samples, are summarized in Table 1.

**Table 1:** Profile of study participants: demographic, clinical, and laboratory parameters

Characteristic	Full Cohort	Sub-Cohort
N	582	234
Age, y; median (IQR)	11 (7–14)	11 (7–15)
Females, n (%)	292 (50.1%)	119 (50.8%)
BMI, kg/m^2^; median (IQR)	19 (15.6–22.7)	18 (16.0–19.75)
Family history of HTN, n (%)	95 (19.8%)	39 (16.6%)
Family history of High cholesterol, n (%)	109 (18.7%)	26 (11.11%)
Family history of Heart Attack, n (%)	15 (2.57%)	5 (2.13%)
Family history of DM, n (%)	103 (17.6%)	30 (12.8%)
Family history of Obesity, n (%)	78 (13.4%)	26 (11.1%)
Family history of Smoking, n (%)	113 (19.4%)	45 (19.23%)
Consanguinity, n (%)	211 (37.9%)	39 (16.6%)
**Traditional biomarkers**		
HbA1c%; (Ref 4.4–6.4)	5.24 (4.97–5.46)	5.22 (4.95–5.44)
Glucose, mmol/L; (Ref 3.9–6.1)	4.86 (4.49–5.36)	4.81 (4.47–5.28)
HsCRP; mg/L; (Ref < 5)	0.53 (0.21–1.6)	0.33 (0.16–0.65)
Total Cholesterol mmol/L; (Ref 3.1–5.6)	4.05 (3.6–4.5)	4.0 (3.60–4.5)
HDL mmol/L; (Ref 0.91–2.21)	1.39 (1.17–1.64)	1.48 (1.27–1.75)
LDL mmol/L; (Ref 2.6–3.3)	2.47 (2.08–2.9)	2.41 (2.06–2.82)
Triglycerides mmol/L; (Ref 0.4–2.1)	0.81 (0.61–1.17)	0.73 (0.59–1.0)

Biomarkers are expressed as Median (IQR). HDL, high-density lipoprotein cholesterol; Hs CRP, high-sensitivity C reactive protein; IQR, interquartile range; LDL, low-density lipoprotein cholesterol; Ref, reference range. TG, triglyceride

### Liquid chromatography with tandem mass spectrometry (LC-MS/MS)

#### Materials

Analytical grade chemical solvents, standards, and other materials were purchased from different companies. The powder forms of CerC16:0 [C16:0-ceramide (d18:1/16:0)], CerC18:0 [C18:0-ceramide (d18:1/18:0)], CerC22:0 [C22:0-ceramide (d18:1/22:0)], CerC24:0 [C24:0-ceramide (d18:1/24:0)], CerC24:1 [C24:1-ceramide (d18:1/24:1(15Z))] were purchased from Avanti Polar Lipids [Alabaster, AL]. The powder forms of Cer internal standards CerC16:0-d7 [C16:0-ceramide-d7 (d18:1-d7/16:0)], CerC18:0-d7 [C18:0-ceramide-d7 (d18:1-d7/18:0)], CerC24:0-d7 [C24:0-ceramide-d7 (d18:1-d7/24:0)], CerC24:1-d7 [C24:1-ceramide-d7 (d18:1-d7/24:1(15Z))] were also purchased from Avanti Polar Lipids [Alabaster, AL]. Albumin from human serum, phosphate-buffered saline, ammonium formate, and formic acid for LC-MS were obtained from Sigma-Aldrich [Burlington, Massachusetts, United States]. LC-MS-grade methanol, LC-MS-grade water, LC-MS-grade acetonitrile, and LC-MS-grade 2-propanol were purchased from Merck [United States]. Ethyl acetate and hexane were purchased from Fisher Scientific [Hampton, New Hampshire, United States]. The Acquity Ultra Performance Liquid Chromatography (UPLC) Ethylene Bridged Hybrid (BEH) C18 column (2.1 mm x 50 mm x 1.7 μm) was obtained from Waters [Milford, Massachusetts].

### Stock and working standard solutions preparation

The powder form of each Cer and corresponding internal standard were dissolved in chloroform: methanol (50:50) to prepare the stock solutions with a concentration of 5 mg/ml and kept at -20℃. The working solution or the desired Cer concentration was prepared from stock solution by serial dilution in methanol. The calibrants of the calibration curve and quality controls for the mixture of Cer were prepared in a surrogate matrix comprising a combination of albumin from human serum and phosphate-buffered saline. To attain the targeted concentrations, the stock solutions were mixed and diluted with the surrogate matrix to formulate a set of eight calibrants for the calibration curve and four levels of Quality Controls (QCs), namely Quality Control High (QCH), Quality Control Medium (QCM), Quality Control Low (QCL) and Lower Limit of Quantification (LLOQ).

### Sample extraction

The plasma samples for children, which were previously frozen, were removed from the − 80 ℃ refrigerator and left to thaw for around 25 min at room temperature. The plasma samples were subjected to vortexing, and then 250 µl of every sample was relocated into a new glass tube to initiate a liquid-liquid extraction process. A 50 µl cocktail of the Cer internal standards CerC16:0-d7, CerC18:0-d7, CerC24:0-d7, and CerC24:1-d7 was added to all calibrants, QC’s, and samples, the internal standard for CerC22:0 was not available from the company, which we have purchased the Cer from it, so we used CerC24:0-d7 as an internal standard for CerC22:0. A mixture of 1 ml of hexane and ethyl acetate (9:1) was added to extract the Cer from the samples. The resulting solution was subjected to vortexing for a few minutes. The mixture was centrifuged for 20 min at room temperature at a speed of 2800 x g. The supernatant layer was isolated and moved to a new test tube, and an additional extraction was conducted on the remaining lower layer. All the extracted layers were combined and dried at room temperature using a gentle stream of nitrogen gas in a sample concentrator. Finally, a mixture of water, acetonitrile, and isopropanol (5%: 47.5%: 47.5%) of LC-MS/MS grade was added to reconstitute the residue.

### Instrumentation

**Fig. 1:**
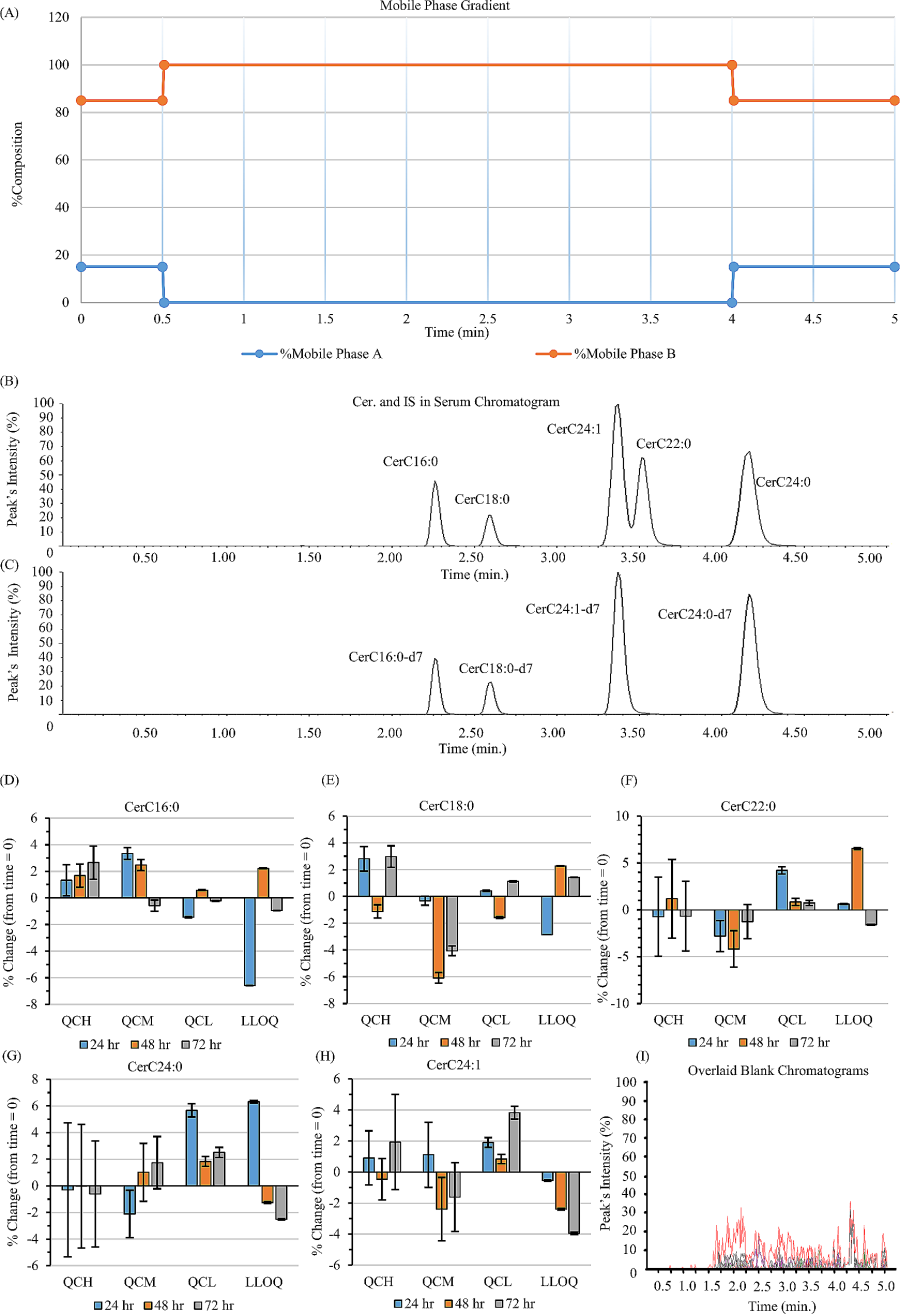
Integrated chromatograms, stability, and specificity of the liquid chromatography-mass spectrometry (LC-MS/MS) ceramide quantification assay. During the 5-minute run, Mobile Phase A comprised 10 mM ammonium formate and 0.1% formic acid in LC-MS grade water. Mobile Phase B contained 10 mM ammonium formate and 0.1% formic acid in LC-MS grade water/acetonitrile/isopropanol (5%: 47.5%: 47.5%) **(A)**. Ceramide metabolites and internal standards were separated adequately based on molecular weights and polarities (**B**-**C**). The stability of ceramide metabolites over three freeze/thaw cycles showed percentage changes represented by blue, orange, and grey bars after 24, 48, and 72 h, respectively. Quality control levels confirmed percentage changes within ± 7% for all metabolites CerC16:0 (**D**), CerC18:0 (**E**), CerC22:0 (**F**), CerC24:0 (**G**), and CerC24:1 (**H**). Overlaid chromatograms of blank samples (**I**) confirmed method specificity, showing no interfering peaks at the respective retention times for ceramide metabolites. IS, Internal Standard; %change, stability

The Ultra High-Pressure Liquid Chromatography-Tandem Mass Spectrometry System (UHPLC-MS/MS) is composed of a Waters Acquity UPLC® I class Plus system which consists of a Binary Solvent Manager (BSM), a Column Manager (CM), and a Sample Manager with flow through needle (SM-FTN) coupled with Triple Quadrupole Mass Spectrometer [Xevo TQ-S micro (Waters, Milford, USA)]. The UHPLC system’s enhanced pressure tolerance allows for the utilization of a narrow-diameter column and fine particle size. The reversed phase analytical column mentioned in the Materials section separated the Cer. A guard column was connected to it to provide physical filtration and prolong the lifespan of the analytical column. 2 µl of the sample was injected into the UHPLC-MS/MS system for quantitative and qualitative analysis. A wash program was employed to clean the needle before and after each injection using acetonitrile/water (75:25, v/v) to reduce carryover and sample contamination. The flow rate and the column’s temperature were maintained at 0.3 ml/min and 40 ℃, respectively. During instrument analysis, two different mobile phases were employed. Mobile phase A comprised LC-MS grade water, and mobile phase B was a mixture of LC-MS grade water/acetonitrile/isopropanol (5%: 47.5%: 47.5%). Both mobile phases contained 0.1% formic acid and 10 mM ammonium formate. The ideal separation of Cer metabolites was accomplished by employing a binary mobile phase gradient pump with the following configuration: from time 0 to 0.5 min, the percent composition of mobile phases A and B was set at 15% and 85%, respectively, followed by an isocratic step at 100% B until 4.0 min. For the equilibration stage, the mobile phases A and B were reset at 15% and 85% for one minute (Fig. 1A).

The ion source used in the MS/MS was Electrospray Ionization (ESI), and it was set in positive mode for all tested Cer. The following MS/MS conditions, such as Nebulizing Nitrogen Gas Flow, Desolvation Temperature, and Desolvation Gas Flow, were used to attain the optimal Multiple Reaction Monitoring (MRM) transitions for Cer, and they were set at 7 L/minute, 500 ℃, and 1000 L/hour, respectively. The direct infusion of individual Cer standards at 1 µg/ml concentration at a flow rate of 20 µl/min was performed to optimize the MRM transitions for Cer. The Masslynx software was employed to manage the UHPLC and MS/MS conditions and was used to analyze or handle the data.

### Method validation

According to the United States Food and Drug Administration (FDA) guidelines, the bioanalytical method should be validated for accuracy, precision, linearity, recovery, specificity, and stability [14, 15]. The determination of bioanalytical parameters was evaluated by analyzing four levels of QC’s (QCH, QCM, QCL, and LLOQ).

#### A. Calibration curves, quality controls and Linearity

The calibration curve’s linearity was expressed by the square of the correlation coefficient (R^2^) of the linear regression curve, and it was ascertained using eight concentration levels for all examined Cer. The calibration curve was plotted using the peak area ratio of Cer to its internal standard versus the Cer concentration. The quality controls were prepared and analyzed to test the validity of the calibration curve.

#### B. Intra-day and inter-day precision and accuracy assay

The percent coefficient of variation (CV%) of several measurements is known as precision. In contrast, the percent error (PE%) of the measured value to the nominal value is expressed as accuracy [14, 16]. The inter and intra-day accuracy and precision of Cer assay were determined by analyzing four levels of quality controls (QCH, QCM, QCL, and LLOQ) in six replicates at each level in each validation run. The mean and standard deviation values of six replicates of quality controls at each stage were used to calculate the Intra-day and Inter-day precision and accuracy as in the following equations:

$$\% {\rm{CV}}\, = \,\frac{{\left( {standard\,Deviation} \right)}}{{\left( {Mean} \right)}}\,x\,100$$

$$\% {\rm{PE}}\, = \,\frac{{\left( {mean\,value} \right)}}{{\left( {nominal\,value} \right)}}\,x\,100$$

#### C. Extraction recovery

The percentage of the known analyte concentration retrieved throughout the sample extraction process is known as extraction recovery. It was evaluated by comparing two different sets of quality controls. The first set consisted of six replicates of QC samples for each level (QCH, QCM, QCL, and LLOQ) spiked with methanol, while the second set was spiked with a blank surrogate matrix, and the extraction was carried out for the second set. After that, the instrument analyzed the two sets, and the area under the peak in the chromatogram was used to compare the two sets to calculate recovery. The following equation was used to calculate recovery:

$${\rm{Recovery\% }}\, = \,\frac{{\left( {mean\,extracted\,QC\,values} \right)}}{{\left( {mean\,unextracted\,QC\,values} \right)}}\,x\,100$$

#### D. Sensitivity

The lowest analyte concentration that can be detected and quantified is termed sensitivity. The Limit of Detection (LOD) and LLOQ can evaluate the sensitivity. The LOD was established by serially reducing the analyte concentration until the signal response was equal to three times the background noise level (Signal-to-Noise [S/N] ratio = 3). The LLOQ was determined when at least S/N ratio = 10.

#### E. Stability and specificity

The percentage of intact analyte in each matrix under particular use and storage conditions compared to the initial concentration throughout time is referred to as the stability (percentage change) [16]. A stability test was conducted by analyzing six replicates of QC samples at four levels (QCH, QCM, QCL, LLOQ), and these spiked QCs underwent three freeze/thaw cycles at 0 h, 24 h, 48 h, and 72 h. The results of QC sample analyses at four concentration levels over 24 h, 48 h, and 72 h were compared from time zero to assess the percent change of all Cer.

The specificity was determined by analyzing six replicates of blanks. The chromatograms of these blanks were overlaid to observe if there were any coeluting or interfering peaks that could interfere with the peaks of targeted analyte species.

### Lipoprotein-a measurement

Lipoprotein-A was measured in the entire cohort (*n* = 582) using the Human Lipoprotein-A Matched Antibody Pair Kit from Abcam (ab217612). A 96-well microplate was used, with each well initially coated with 50 µL of a 2 µg/mL capture antibody, followed by an overnight incubation at 4 °C. Subsequent steps included washing the plate with 350 µL of Wash Buffer blocking with 300 µL of blocking buffer for 2 h at room temperature to reduce non-specific binding and additional washing. Serially diluted standards were prepared from 1300 ng/ml to 0 ng/ml. Then, 50 µL of diluted sample/standard solutions were added to the plate and incubated for 2 h at room temperature.

Further washing was performed, and 50 µL of detector antibody diluted in blocking buffer was added to each well, followed by incubation. After additional washing, 50 µL of 1X HRP-conjugated streptavidin solution was added and incubated. TMB substrate (100 µL) was added to each well and set for up to 20 min in the dark. The reaction was stopped with 100 µL of stop solution, and the plate was mixed. OD at 450 nm was recorded. The target protein concentration in the sample was determined by interpolating the blank control subtracted absorbance values against the standard curve, considering the appropriate sample dilution factor.

### Defining the reference population and intervals

To establish reference intervals for the examined Cer species, we retrospectively identified a subgroup from the original pool of 582 study participants, adhering to the criteria outlined by the International Federation of Clinical Chemistry (IFCC) Committee on Reference Intervals and Decision Limits and Centers for Disease Control and Prevention (CDC) [17, 18]. A total of 234 participants were selected based on their adherence to the IFCC recommendation and the calculations of Age for weight Z-score and BMI for Age Z-score (Range from − 1 to 1) in alignment with guidelines from the CDC. These 234 healthy individuals (sub-cohort), aged between 6 and 17 years, were utilized to determine the reference range for Cer levels.

Reference intervals for Cer levels were calculated using Bertholf’s method [19–21], mainly by sorting the nonparametric data in ascending order and excluding the upper and lower 2.5% of the values. Each bootstrap sample is created by randomly selecting observations from the original dataset with replacement, allowing data points to be chosen more than once. This resampling procedure has been repeated many times, typically 1,000 iterations in our study. Then, the reference range was calculated using the nonparametric rank percentile method for each bootstrap sample. The resulting range is defined by the values that remain within these limits, encompassing the central 95% of the dataset.

### Ceramide risk score calculation

The Cer risk score (CERT-1) was calculated as described [22]. CerC16:0, CerC18:0, and CerC24:1 concentration and their respective ratios to CerC24:0 were estimated for every participant. Then, they were compared with the whole study cohort (*n* = 582). If the variable lies between the 3rd and 4th quartiles, the individual receives + 1 and + 2 points, respectively. This process is repeated for each type of the Cer mentioned above and their respective ratios to CerC24:0; thus, the score ranges from 0 to 12. Based on this score, the subjects were divided into four risk groups (0–2, 3–6, 7–9, and 10–12). The higher the score the participant has, the higher the risk of developing CMD in the future. Box-Cox transformation was applied to concentrations to adjust for non-normal distribution and stabilize the variance.

### Statistical analysis

Statistical analyses were implemented with SPSS 27.0 (IBM, NY, USA) and R Studio software version 4.2.1 with a significant *P* value < 0.05. Continuous variables were tested for normal distribution using Shapiro-Wilk and Kolmogorov-Smirnov tests. Nonparametric data were expressed as median and interquartile range, while parametric values were reported as Mean ± SEM. Bivariate correlations and multiple regression analysis were conducted to study the relationship between different Cer concentrations, CERT-1 score, and the traditional risk factors for CMD, such as total cholesterol, triglyceride, low-density lipoprotein, inflammatory cytokines (Interleukin-6, Tumor Necrosis Factor-α) and high-sensitivity C-reactive protein.

## Results

Development of a sensitive and specific Ultra-High-Pressure Liquid Chromatography-Tandem Mass Spectrometry system (UHPLC-MS/MS) assay for simultaneous quantification of five cardiometabolic disease-risk-predicting ceramide species.

### Assay conditions

Five Cer were separated in the UHPLC based on their retention times, molecular weights, and polarities, as shown in Fig. 1B-C. Under the applied ESI source, the Cer generate the optimal transitions of [M + H-18] ^+^ to 264 m/z (mass-to-charge ratio). For example, the MRM transitions of CerC16:0, CerC18:0, CerC22:0, CerC24:0, and CerC24:1 were 520.6 > 264.3, 548.6 > 264.3, 604.6 > 264.3, 632.6 > 264.3, and 630.6 > 264.3 respectively as illustrated in Table 2. The internal standard of Cer generates product ion spectra comparable to the main Cer because it yields the expected 7 Da mass shift in the main Cer. For instance, the MRM transitions for CerC16:0-d7, CerC18:0-d7, CerC24:0-d7, and CerC24:1-d7 were 527.5 > 271.3, 555.6 > 271.3, 639.7 > 271.3, and 637.4 > 271.3; respectively (Table 2). The optimal collision energies (eV) values used to attain these MRM transitions are illustrated in Table 2.

**Table 2:** The Multiple Reaction Monitoring (MRM) parameters of ceramide metabolites and corresponding internal standards

No.	Ceramide (Cer)	Mass (g/mol)	Precursor (m/z)	*Product (m/z)	Collision energy (eV)
1	CerC16:0	537.901	520.59	264.33 282.34 252.31	20 20 24
2	CerC18:0	565.95	548.57	264.33 282.34 252.31	22 24 26
3	CerC22:0	622.06	604.62	264.33 282.34 252.31	24 26 30
4	CerC24:0	650.11	632.67	264.33 282.34 252.31	34 30 30
5	CerC24:1	648.09	630.655	264.33 282.34 252.31	28 28 30
6	CerC16:0-d7	544.94	527.51	271.3 289.36 259.28	22 22 26
7	CerC18:0-d7	572.99	555.62	271.29 289.36 259.34	22 24 24
8	CerC24:0-d7	657.156	639.72	271.29 289.35 259.33	30 30 32
9	CerC24:1-d7	655.14	637.45	271.35 289.35 259.33	28 26 26

*The ceramide generate the optimal transitions of [M + H-18] ^+^ to 264 m/z (mass-to-charge ratio)

CerC16:0 [C16:0-ceramide (d18:1/16:0)]; CerC18:0 [C18:0-ceramide (d18:1/18:0)]; CerC22:0 [C22:0-ceramide (d18:1/22:0)]; CerC24:0 [C24:0-ceramide (d18:1/24:0)]; CerC24:1 [C24:1-ceramide (d18:1/24:1(15Z))]; CerC16:0-d7 [C16:0-ceramide-d7 (d18:1-d7/16:0)]; CerC18:0-d7 [C18:0-ceramide-d7 (d18:1-d7/18:0)]; CerC24:0-d7 [C24:0-ceramide-d7 (d18:1-d7/24:0)]; CerC24:1-d7 [C24:1-ceramide-d7 (d18:1-d7/24:1(15Z))

### Assay validation

The linear range for the calibration curve was 0.0036 to 0.46 µM for CerC16:0, 0.0021 to 0.27 µM for CerC18:0, 0.012 to 1.54 µM for CerC22:0, CerC24:0, and CerC24:1 (Table 3). The linearity R^2^ was 0.996 for CerC18:0, 0.997 for CerC16:0, 0.998 for CerC22:0, CerC24:0, and CerC24:1 (Table 3). The concentration of QCH, QCM, QCL, and LLOQ for CerC22:0, CerC24:0, and CerC24:1 was 0.96, 0.23, 0.06, and 0.012 µM, respectively while the concentration of QC’s for CerC16:0, and CerC18:0 was 0.27/0.16 for QCH, 0.07/0.04 for QCM, 0.02/0.0099 for QCL, and 0.0036/0.0021 µM for LLOQ, respectively (Table 3).

**Table 3:** The liquid chromatography-mass spectrometry (LC-MS/MS) ceramide quantification assay validation results

N	Ceramide	*QC samples	Concentration (µM)	Inter-day			Intra-day			Recovery (%)	LOD (µM)	R^2^	Linear Range (µM)
				Precision (CV%)	Accuracy (PE%)	SD	Precision (CV%)	Accuracy (PE%)	SD				
1	CerC16:0	QCH	0.27	3.09	87.93	4.07	1.90	87.27	2.49	64.82	0.00016	0.997	0.0036–0.46
		QCM	0.07	4.08	98.89	1.51	4.78	97.23	1.74	47.46			
		QCL	0.02	2.01	100.42	0.19	0.98	101.14	0.09	51.48			
		LLOQ	0.0036	4.21	111.41	0.09	2.52	114.70	0.06	68.62			
2	CerC18:0	QCH	0.16	3.97	89.79	3.21	2.35	88.38	1.87	66.37	0.00034	0.996	0.0021-0.27
		QCM	0.04	5.23	100.03	1.18	6.87	100.19	1.55	44.12			
		QCL	0.0099	2.98	99.50	0.17	4.12	99.29	0.23	39.97			
		LLOQ	0.0021	2.68	100.85	0.03	2.46	102.28	0.03	59.31			
3	CerC22:0	QCH	0.96	2.75	88.72	14.63	2.97	89.08	15.87	66.61	0.00045	0.998	0.012–1.54
		QCM	0.23	4.05	93.89	5.70	4.58	95.30	6.55	44.12			
		QCL	0.06	3.64	98.89	1.35	3.48	96.74	1.26	35.20			
		LLOQ	0.012	2.32	100.73	0.18	1.60	100.43	0.13	52.62			
4	CerC24:0	QCH	0.96	3.28	88.64	17.42	3.59	88.79	19.10	66.05	0.00045	0.998	0.012–1.54
		QCM	0.23	4.37	94.15	6.16	5.15	95.21	7.36	43.53			
		QCL	0.06	4.52	99.52	1.69	3.84	96.69	1.39	36.59			
		LLOQ	0.012	3.97	106.53	0.33	2.19	103.37	0.18	54.44			
5	CerC24:1	QCH	0.96	1.15	87.23	6.04	0.86	86.78	4.48	60.37	0.00045	0.998	0.012–1.54
		QCM	0.23	5.07	95.09	7.23	5.91	94.54	8.38	40.01			
		QCL	0.06	3.11	93.52	1.09	3.74	92.57	1.30	43.51			
		LLOQ	0.012	2.14	101.42	0.17	2.04	101.71	0.16	52.75			

*Ceramide (Cer) quality control samples (QC) at four levels (QCH [Quality Control High]; QCM [Quality Control Medium]; QCL [Quality Control Low]; LLOQ [ Lower Limit of Quantification])

CerC16:0 [C16:0-ceramide (d18:1/16:0)]; CerC18:0 [C18:0-ceramide (d18:1/18:0)]; CerC22:0 [C22:0-ceramide (d18:1/22:0)]; CerC24:0 [C24:0-ceramide (d18:1/24:0)]; CerC24:1 [C24:1-ceramide (d18:1/24:1(15Z))]; LOD [Limit of Detection]; SD [Standard Deviation]; CV% [percent coefficient of variation]; PE% [percent error]; R^2^ [square of the correlation coefficient]

Generally, the CV percentage was below 7%, while the PE percentage was between 85% and 114% for all Cer (Tables 3 and 4). The percentage recovery for the QC samples ranged between 35% and 68% (Tables 3 and 4). In this study, the LOD ranged from 0.00016 to 0.00045 µM, while the LLOQ ranged from 0.0021 to 0.012 µM (Tables 3 and 4). Our results show that the percentage change of all Cer is within ± 7% over three freeze/thaw cycles at 24 h, 48 h, and 72 h (Fig. 1D-H). The method in this study was specific as it did not observe any coeluting or interfering peaks that could interfere with the targeted analyte (Fig. 1I).

**Table 4:** Comparative results of the liquid chromatography-mass spectrometry (LC-MS/MS) ceramide quantification assay in this study and other relevant studies

Reference	Ceramide	LOD (µM)	LLOQ (µM)	Inter-day Precision (%CV)	Intra-day Precision (%CV)	Inter-day Accuracy (%PE)	Intra-day Accuracy (%PE)	Extraction Method		% Recovery	Injection Volume (µl)	R^2^
								Type	Extraction Solvent			
This Study	CerC16:0	0.00016	0.0036	2.0–4.2	0.9–4.7	87.9–111.4	87.2–114.7	LLE	C_6_H_14_/ EtAc	47.4–68.6	2	0.997
	CerC18:0	0.00034	0.0021	2.6–5.2	2.3–6.8	89.7–100.8	88.3–102.2			39.9–66.3		0.996
	CerC22:0	0.00045	0.012	2.3–4.0	1.6–4.5	88.7–100.7	89.1–100.4			35.2–66.6		0.998
	CerC24:0	0.00045	0.012	3.2–4.5	2.1–5.1	88.6–106.5	88.8–103.4			36.5–66.0		0.998
	CerC24:1	0.00045	0.012	1.1–5.0	0.8–5.9	87.2–101.4	86.8–101.7			40.0–60.3		0.998
Jiang et al. (25)	CerC22:0	–	0.032	1.8–3.2	1.4–3.4	91.1–99.8	90.2–100	PPT	IPA/CHCl3	109	5	0.999
	CerC24:0	–	0.12	1.2–3.6	0.5–3.4	93.2–99.6	92.5–99.8			114		0.997
Kauhanen et al. (24)	CerC16:0	–	0.08	7–8.9	5.9–6.8	97.8–99.8	97–99.5	PPT	IPA/EtAc	99.9–102.1	5	0.992
	CerC18:0	–	0.03	6.5–10.9	4.2–8.2	90.1–95.8	85.1–90.7			100.9 − 106.6		0.993
	CerC24:0	–	0.86	6.6 − 11.8	5.0–6.3	91.4–96.5	86.9–90.5			98.8–99.5		0.998
	CerC24:1	–	0.29	5.7–10	3.7–6.8	94.8–97.5	89.4–92.9			102.1 − 109.1		0.996
Huang et al. (16)	CerC16:0	–	0.005	1.5–6.2	2.4–6.1	96.4–99.7	94.7–99.1	PPT	MeOH	102.3 − 104.5	10	0.999
	CerC18:0	–	0.00099	2.8–6.9	3.8–5.4	93.5–97.6	94.4–99.8			97.2–99.2		0.999
	CerC22:0	–	0.005	1.9–3.3	3.9–5.9	95.9–98.5	91.2–99.6			98.9–101.7		0.999
	CerC24:0	–	0.005	1.7–6.2	2.9–5.4	96.5–98.6	95.1–99.9			99.6–100.2		0.999
	CerC24:1	–	0.005	1.4–4.8	2.6–4.7	95.2–99.1	96–99.7			98.5–103.2		0.999
Basit et al. (27)	CerC16:0	–	0.00098	1.6–3.9	3.0–6.7	92.6–99.6	96.1–99.6	LLE (BD)	CHCl_3_/MeOH	87.1–89.4	3	0.991
	CerC18:0	–	0.00099	0.5–2.6	2.6–7.5	92.5–96.9	93.1–98.9			85.9–91.1		0.993
	CerC22:0	–	0.001	3.2–4.2	2.0–7.9	94.5–99.7	95.6–97.3			87.1–91.1		0.995
	CerC24:0	–	0.001	5.3–7.8	0.7–11.4	95.8 − 100.6	95.5–97.3			87.3–89.7		0.995
	CerC24:1	–	0.00099	1.8–5.6	1.3–8.2	98.5 − 105.4	100.1 103.2			86.1–102.8		0.996
Begou et al. (28)	CerC16:0	0.0013	0.0043	6.2–8.8	1.4–4.1	98.1–103	97.6–103	LLE (MTBE; modified Folch)	MTBE/MeOH; CH_2_Cl_2_/MeOH	66.1–120	5	0.999
	CerC18:0	0.0012	0.0041	6.6–10.8	3.3–5.4	87.8–101	86.6–101.1			82–141		0.998
	CerC24:0	0.00063	0.0022	5.9–14.0	2.2–4.2	94–106	90.1–105.5			104–220		0.999
	CerC24:1	0.00063	0.0022	5.6–11.1	1.8–8.8	91–103	93–101.5			102–145		0.998

CerC16:0 [C16:0-ceramide (d18:1/16:0)]; CerC18:0 [C18:0-ceramide (d18:1/18:0)]; CerC22:0 [C22:0-ceramide (d18:1/22:0)]; CerC24:0 [C24:0-ceramide (d18:1/24:0)]; CerC24:1 [C24:1-ceramide (d18:1/24:1(15Z))]; LLOQ [ Lower Limit of Quantification]; LOD [Limit of Detection]; CV% [percent coefficient of variation]; PE% [percent error]; LLE [liquid-liquid extraction]; PPT [precipitation]; BD [Bligh and Dyer]; MTBE [methyl tert-butyl ether]; MeOH [methanol]; CHCl_3_ [chloroform]; IPA [isopropanol]; EtAc [ethyl acetate]; C_6_H_14_ [hexane]; CH_2_Cl_2_ [dichloromethane]; R^2^ [square of the correlation coefficient]

### Establish reference range values for assessing cardiometabolic disease risk using ceramide levels in individuals under 18

The 2.5th and 97.5th percentiles for plasma levels of CerC16:0, CerC18:0, CerC22:0, CerC24:0, and CerC24:1 have been determined as a double-sided reference interval in the healthy cohort of 234 selected participants aged 6–17 years (Table 5). The reference values specific to this study population were set to be for CerC16:0 (0.12–0.29 µmol/L), CerC18:0 (0.019–0.067 µmol/L), CerC22:0 (0.102–0.525 µmol/L), CerC24:0 (0.65–1.54 µmol/L), and CerC24:1 (0.212–0.945 µmol/L). The average ratios of normal young individuals for CerC16:0, CerC18:0, and CerC24:1 to CerC24:0 were estimated to be < 0.265, < 0.056, and < 0.988, respectively (Table 5). Additionally, the expected normal ratios of these Cer to CerC22:0 were estimated and set to be < 1.534 for CerC16:0/CerC22:0, < 0.253 for CerC18:0/CerC22:0, and < 2. 28 for CerC24:1/CerC22:0.

**Table 5:** Median and 95% reference ranges of plasma ceramide levels and ratios in individuals aged 6–17 years

Ceramide (Cer)	Median (µmol/L)	IQR	Reference Interval (µmol/L)	Lower Ref Lim 95% CI*	Upper Ref Lim 95% CI*
CerC16:0	0.189	0.162–0.22	0.122–0.297	0.115–0.131	0.283–0.319
CerC18:0	0.035	0.029–0.044	0.019–0.067	0.018 − 0.012	0.065–0.068
CerC24:0	1.159	0.941–1.356	0.65–1.54	0.583–0.697	1.535–1.673
CerC24:1	0.384	0.312–0.478	0.212–0.945	0.201–0.230	0.765–1.155
CerC22:0	0.243	0.185–0.313	0.102–0.525	0.096–0.118	0.454–0.553
CerC16:0/ CerC24:0	0.168	0.144–0.196	< 0.265	-	0.262–0.276
CerC18:0/ CerC24:0	0.031	0.026–0.038	< 0.056	-	0.049–0.059
CerC24:1/ CerC24:0	0.324	0.277–0.399	< 0.988	-	0.846–1.069
CerC16:0/ CerC22:0	0.782	0.643–0.995	< 1.534	-	1.358–1.931
CerC18:0/ CerC22:0	0.149	0.122–0.184	< 0.253	-	0.246–0.262
CerC24:1/ CerC22:0	1.307	1.01–1.59	< 2.28	-	2.117–2.682

^*^Bias-Corrected and accelerated (BCa) confidence intervals for 2.5th -97.5th percentiles; results are based on 1000 bootstrap samples, CI, confidence intervals; IQR; interquartile range; Ref Lim; reference limit

**Fig. 2:**
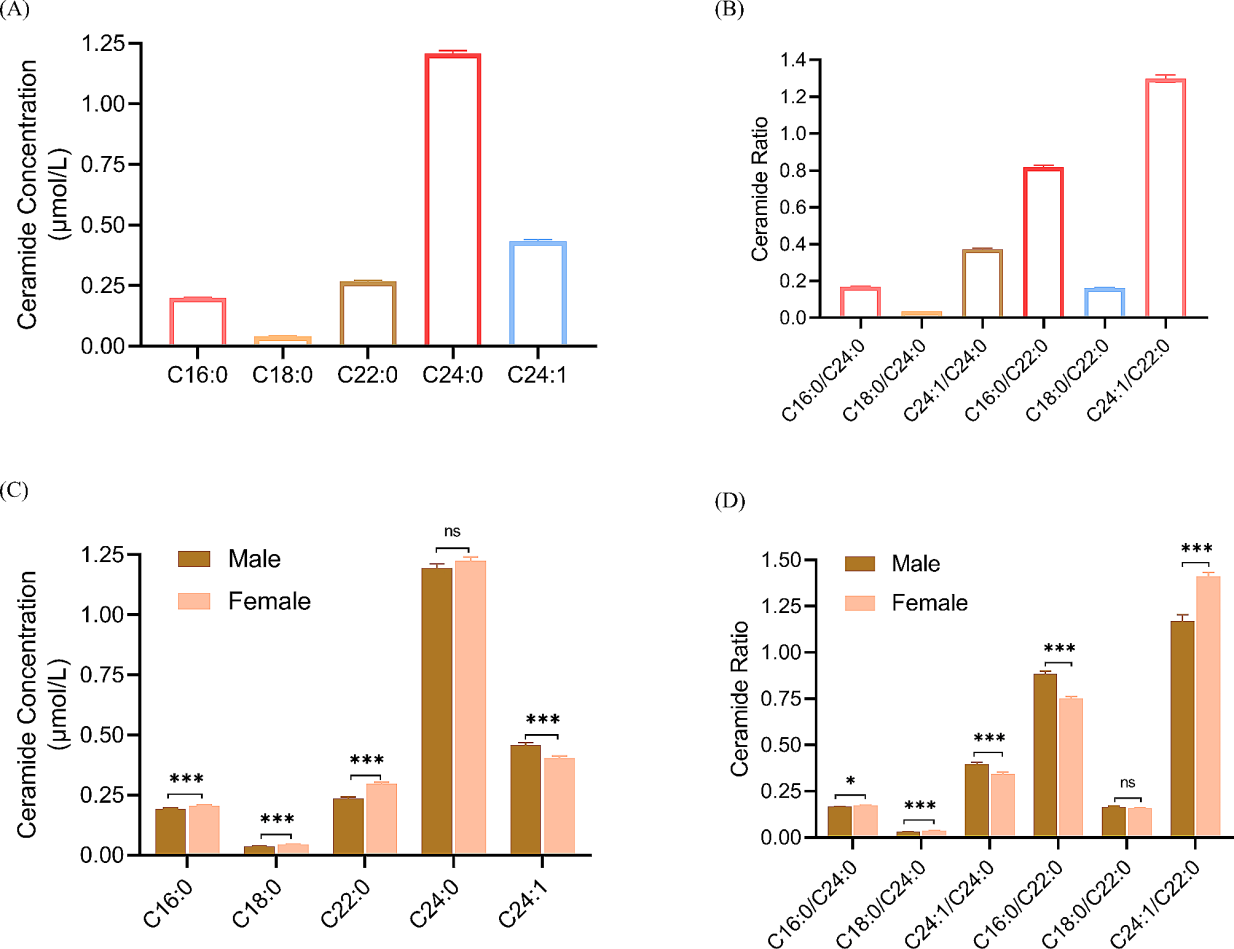
The differential abundance of plasma ceramide species. In plasma, the distribution of ceramides is relatively fluctuating, with C24:0 being the most abundant and C18:0 the least concentration (**A**). Likewise, C24:1/C22:0 and C18:0/C24:0 are the highest and lowest ratios of plasma ceramide, respectively (**B**). Independent two-tailed t-test has shown a significant difference between males and females in all measured ceramide species, except for the concentration of C24:0 (ns; not significant *P* > 0.05) (**C**), and the ratio of C18:0/22:0 (*P* > 0.05) (**D**). Data are presented as Mean ± SEM

To investigate the variance in the abundance of measured Cer species in plasma, we measured the exact concentration of the five Cer in an additional 348 participants under 18. This expanded the overall cohort size to 582 participants (Table 1). In plasma, CerC24:0 was the most abundant Cer (1.21 ± 0.012 µmol/l), followed by CerC24:1 (0.43 ± 0.007 µmol/l), then CerC22:0 (0.27 ± 0.004 µmol/l) (Fig. 2A; Mean ± SEM), in agreement with our previously findings [10].

Apart from CerC24:0 which revealed no significant difference between females and males [1.22 ± 0.01 vs. 1.19 ± 0.017 µmol/l (*p* =0.21)], two-tailed independent t-test showed that females (*n* = 290) had significantly higher concentrations of all measured Cer versus males (*n* = 284); CerC16:0 [0.205 ± 0.002 vs. 0.19 ± 0.003 µmol/l (*p* <0.05)], CerC18:0 [0.044 ± 0.008 vs. 0.037 ± 0.0009 µmol/l (*p* <0.01)], CerC22:0 [0.2697± 0.005 vs. 0.235 ± 0.006 µmol/l (*p* <0.01)], yet CerC24:1 [0.403 ± 0.008 vs. 0.457 ± 0.009 µmol/l (*p* <0.05)] were higher in males group. Significant differences were observed in CerC16:0/CerC24:0 [0.17 ± 0.002 vs. 0.16 ± 0.002 µmol/l (*p* <0.05)], CerC18:0/CerC24:0 [0.037 ± 0.0006 vs. 0.031 ± 0.006 µmol/l (*p* <0.01)] and CerC24:1/CerC24:0 [0.34 ± 0.008 vs. 0.395 ± 0.01 µmol/l (*p* <0.01)] (Fig. 2B; Mean ± SEM).

### Circulating ceramide levels and the associated CERT-1 risk score provide insights into the potential cardiometabolic risk

We performed correlation analysis to assess the correlations between the measured Cer circulating levels and the traditional CMD risk factor. Our findings showed a strong positive correlation between high-risk Cer, namely CerC16:0 vs. CerC18:0, CerC16:0 vs. CerC24:1, and CerC16:0 vs. CerC24:1 (*Rho* = 0.648, 0.533, 0.45, respectively, *p* < 0.01) in the lipidomic profiling of the samples. Likewise, CerC22:0 and CerC24:0 had a significant positive correlation (*Rho* = 0.7, *p* < 0.01).

Regression analysis was also conducted to study the relativity between Cer species that are used to build CERT-1 score, including CerC16:0 vs. CerC24: 1, CerC18:0 vs. CerC24:1, CerC16:0 vs. CerC18:0, CerC22:0 vs. CerC24:0 in the dataset. Regarding each pair of Cer, the analysis generated regression models exhibiting a significant relationship (*p* < 0.01) with R^2^ values ranging from 0.18 to 0.5, as shown in Supplementary Figure S1. These R² values indicate that around 18% and 14% of the variability in the dependent variable (e.g., CerC24:1) can be explained by the independent variable CerC16:0 and CerC18:0, respectively (Supplementary Figures S1A-B), CerC16:0 based on CerC18:0 explains 38% of the variability (Supplementary Figure S1C) and 50% for the beneficial pair; CerC24:0 vs. CerC22:0 (Supplementary Figure S1D).

**Fig. 3:**
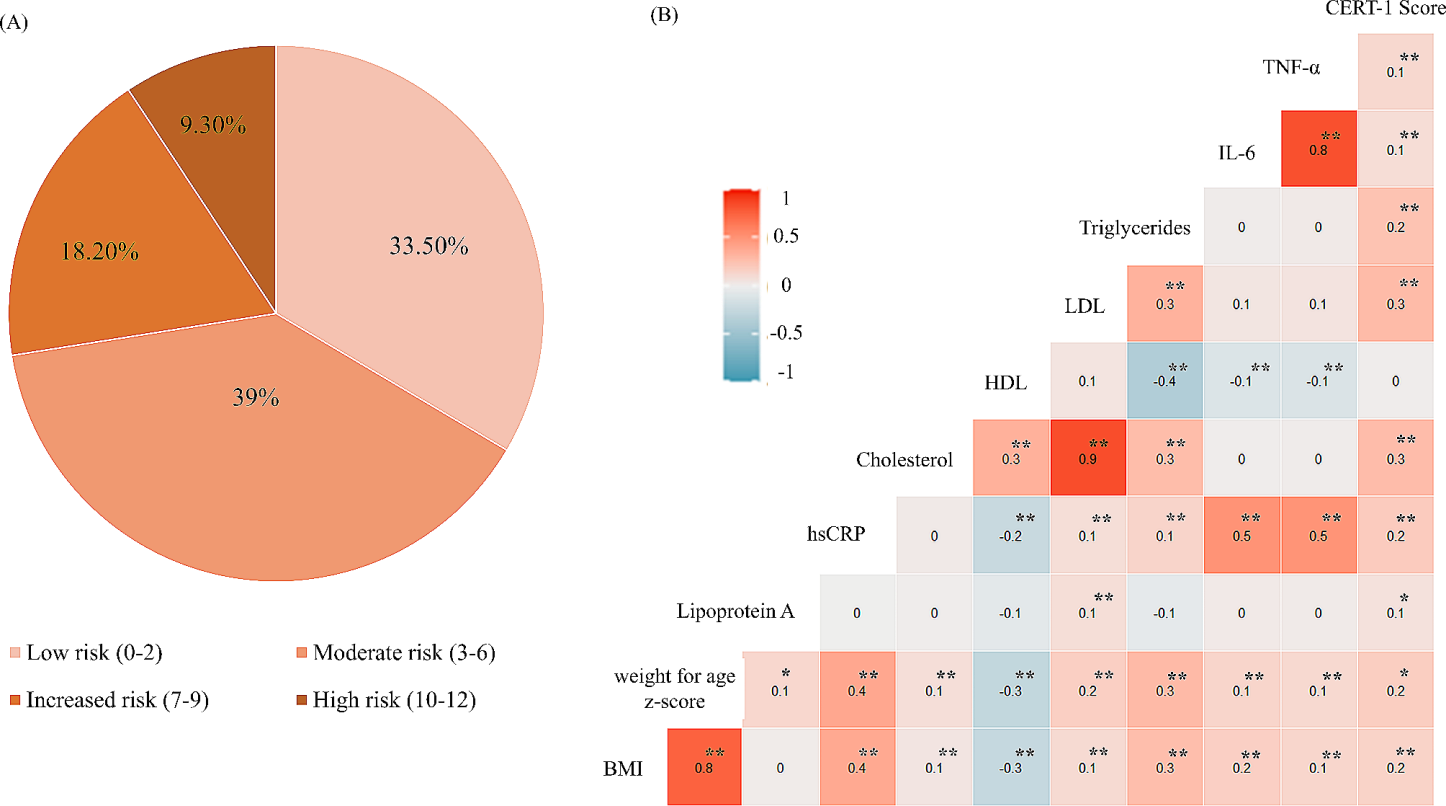
The CERT-1 score stratifies the 582 study participants into distinct risk groups and is correlated with cardiometabolic disease risk factors. The risk stratification analysis of the entire cohort (*n* = 582) using the ceramide-based risk score CERT-1 revealed that most of the study population, comprising two-thirds (72.5%), falls into the low to moderate risk category. A notable proportion, 18.2%, exhibits a relatively increased risk, while 9.3% are identified as being at a higher risk of developing cardiometabolic disease (**A**). Spearman’s rank correlation analysis detected a statistically significant yet mild to moderate positive correlation (ρ = 0.1–0.3, *p* < 0.01) between CERT-1 scores and traditional cardiometabolic biomarkers (**B**). TNF-alpha, Tumor necrosis factor-alpha; IL-6, Interleukin-6; LDL, low-density lipoprotein; HDL, high-density lipoprotein; hsCRP, high-sensitivity C reactive protein; BMI, Body mass index

To evaluate the utility of the CERT-1 score in predicting early risks of cardiometabolic outcomes, including CVD and DM2, in apparently healthy young individuals, we calculated and applied the CERT-1 score using the previously described methodology [22]. The application of the CERT-1 score on this cohort stratified the participants (*n* = 582) into four distinct risk groups, including low-risk (score 0–2; *n* = 195), moderate-risk (Score 3–6; *n* = 227), increased-risk (Score 7–9; *n* = 106), and high-risk (score 10–12; *n* = 54) (Fig. 3A). Furthermore, the CERT-1 score exhibited significant associations with traditional CMD risk factors, including Total Cholesterol, Triglycerides, Low-Density Lipoprotein, Lipoprotein A, BMI, and inflammatory cytokines (IL-6, TNFα, hs-CRP), as illustrated in Fig. 3B.

Multiple regression analysis explored which cardiometabolic risk factors (i.e., Total Cholesterol, hs-CRP, Triglycerides, LDL, Gender) may significantly determine the CERT-1 score as an independent variable. Table 6 shows the results of the multiple linear regression, where the CERT-1 score was included as a dependent variable. The model was highly significant (R^2^ = 0.153; F (5,576) = 20.8; *p* < 0.001). Regression verified the relationship between CMD risk factors and CERT-1 score as several independent predictors: Triglycerides (B = 1.035, *p* < 0.01), Total Cholesterol (B = 0.689, *p* < 0.05), hs-CRP (B = 0.222, *p* < 0.01), and female gender (B = 0.616, *p* < 0.05) were positively related to higher risk outcome, and significantly influenced CERT-1 risk score.

**Table 6:** Multiple regression analysis results of CERT-1 total score in the 582 study participants

Independent variables	Regression coefficient B	Standardized Beta	95% CI of the coefficients	*P* value
Triglycerides	1.035	0.16	0.51 – 1.56	< 0.01
Total Cholesterol	0.689	0.157	0.5 – 1.32	< 0.05
hs-CRP	0.222	0.162	0.12 – 0.33	< 0.01
Gender				
Male	Reference	0.092	0.1 – 1.13	< 0.05
Female	0.616			
LDL	0.399	0.08	-0.33 – 1.12	0.28
Intercept	-0.727		-2.21 – 0.76	0.33
R^2^ = 0.153				

Hs-CRP, high sensitivity C-reactive protein; LDL, low density lipoprotein

## Discussion

Numerous clinical studies have consistently demonstrated that Cer is an independent predictor and robust prognostic biomarker for predicting future CMD and its associated complications, surpassing traditional risk factors [2, 6, 7, 9, 22, 23]. Hence, several research and clinical laboratories compete to develop Cer assays with greater sensitivity, specificity, and throughput from complex biological samples [16, 24–26]. In line with this objective, we have devised and validated a UHPLC-MS/MS assay for quantifying and qualifying five CMD-related Cer species C16:0, C18:0, C22:0, C24:0, and C24:1 in human plasma. The linearity of calibration curves for all Cer in our method was similar to that of calibration curves from previous studies (Table 4) [24, 25, 27, 28]. For all QC’s, the precision and accuracy values were in an acceptable range of ≤ ± 15%, except for LLOQ, which should be no more than ± 20% [14]. The precision and accuracy of our method were comparable to the previous approaches in the earlier studies (Table 4). For example, one of the studies showed the precision ranged from 3.7 to 11.8% [24], while another study illustrated the precision ranged from 1.4 to 14.0% [28]. Our study showed that the precision ranged from 0.8 to 6.8%, reflecting a higher precision of our methodology. The accuracy of our method was almost similar to that of the previous methods (Table 4). Although our recovery percentage was slightly lower compared to the earlier studies listed in Table 4, the recovery of Cer analytes was enough to detect Cer levels in the whole cohort since our technique is very sensitive. Noteworthy, the recovery percentage can be enhanced by performing triple extraction instead of double extraction during sample processing. One study reported that the LLOQ for CerC22:0 and CerC24:0 were 0.032 and 0.12 µM, respectively [25]. In another study, the LLOQ for CerC16:0, CerC18:0, CerC24:0, and CerC24:1 was 0.08, 0.03, 0.86, and 0.29 µM, respectively [24]. Xuefeng et al. reported almost similar LLOQ to our study, which was 0.005 and 0.00099 µM for CerC16:0 and CerC18:0, while 0.005 for CerC22:0, CerC24:0, and CerC24:1, respectively [16]. The LOD in another study ranged from 0.0012 to 0.00063 ng/ml [28], while in our study, the LOD was less than that. The details regarding the sensitivity are found in Table 4. These results emphasize the ultra-sensitivity of the newly developed assay.

This heightened sensitivity provided a foundational cornerstone for the subsequent establishment of reliable reference intervals for Cer concentrations in young individuals for the first time, contributing to a deeper understanding of their role in predicting future health hazards through precise risk assessment.

Prior research studies [16, 24–26] and diagnostic laboratories [12] have primarily investigated and reported reference ranges for Cer in adult individuals (> 18 years old), predominantly focusing on patients with CMD in America or Europe. While reference values can be obtained from foreign commercial manufacturers or scientific literature, various factors, including ethnicity, analytical methods, gender, and age, can influence these values [19, 29–32]. Hence, it has become crucial to identify a healthy cohort within each population and establish reference ranges specifically tailored for CMD-risk ceramide [13]. In this study, we could quantify levels of five Cer (C16:0, C18:0, C22:0, C24:0, C24:1) in a cohort of 234 schoolchildren below the age of 18 to establish reference values for these Cer species within the Emirati population of this specific age group. Aligned with our results, Lopez et al. assessed CerC18:0 and CerC22:0 in a small cohort of 14 lean, healthy females aged 10–17 [23]. In their study, CerC18:0 and CerC22:0 were observed to be within the reference range identified in our investigation.

Although many Cer species are correlated with each other with different strengths [33], strong correlations between high-risk Cer indicate a close interconnection in their metabolic or regulatory pathways, suggesting shared roles in pathological processes. The regression models further support the significance and direction of the relationships among these lipid derivatives, aiding in understanding the dependencies in their occurrence, which can be valuable in clinical and diagnostic contexts. For instance, levels of one Cer may act as predictive or diagnostic markers for another, which, in turn, could facilitate disease diagnosis, particularly in cases where multiple Cer types contribute to the same disease [34]. Previous research has demonstrated that the ratio of high-risk Cer to CerC24:0 and CerC22:0 are robust predictors of CMD risk, preeminent adverse cardiac events (MACE), and death [9–11, 35]. This accumulating evidence has led to their integration into clinical diagnostic laboratories [12].

The Cer-based concentration and clinical scores, including the CERT-1 score, have emerged as novel biomarkers for assessing the onset and adverse events in adult patients with cardiovascular disease and type 2 diabetes [6, 22, 35]. In our study, the CERT-1 score demonstrated significant associations with traditional cardiometabolic risk factors, such as total cholesterol, triglycerides, low-density lipoprotein, lipoprotein A, body mass index, and inflammatory cytokines. This multi-factorial approach to risk assessment enhances the predictive power of the CERT-1 score. Moreover, these findings align with other studies that reported an interrelated role between the increase in plasma Cer, proinflammatory cytokines, and NAFLD. For instance, TNF-α stimulates Cer synthesis through binding to Tumor necrosis factor receptor 1, activating enzyme-generating Cers, namely acid and neutral sphingomyelinases. Besides inducing insulin resistance, this interplay between TNF- α and Cers amplifies mitochondrial reactive oxygen species production, thereby promoting apoptosis. This cascade contributes to increased recruitment of inflammatory cells to the liver, exacerbating hepatic inflammation. Similarly, elevated levels of interleukin-6 showed a notable correlation with plasma Cers and have been linked to the severity of non-alcoholic steatohepatitis [36].

Collectively, these findings highlight the potential value of the CERT-1 score in assessing cardiometabolic risks, as well as its possible use in predicting adverse health outcomes associated with the intricate interplay between proinflammatory cytokines, Cer, and hepatic inflammation.

The significance of this implementation arises not only from its role as an assessment tool but also as a potential therapeutic indicator, supported by various studies demonstrating the treatability of Cer as targets [9, 10].

## Conclusion

This research defined reference ranges for five specific Cer species in plasma samples collected from Emirati participants under 18 years, accomplished through a validated LC-MS/MS assay. These reference intervals provide a crucial foundation for interpreting Cer levels in clinical practice and research endeavors, facilitating the early identification of deviations from normal ranges that may signify increased CMD risk. Although the present study uncovers a correlation between the Cer-based risk score (CERT-1) and cardiometabolic risk factors, it remains a compelling subject for future inquiries to determine whether circulating Cer levels can predict future events in children and contribute to healthcare advancements.

### Limitations

Study limitations include the percentage recovery of the extraction method for plasma Cer, which was relatively low. Additionally, the lack of follow-up data, the limited number of participants, and the corresponding baseline information may be of concern. Consequently, Cer reference intervals and risk scores derived from our study should be further validated within larger cohorts at the same age, encompassing comprehensive demographics and follow-up. This validation is beneficial to confirm the normal range and refine relative risk estimates for various risk categories. Additionally, the relatively young age of the participants in this study may be contributing to somewhat optimistic association results. This optimism might differ in a real-world patient care scenario, where controlling confounding factors is more challenging.

### Electronic supplementary material

Below is the link to the electronic supplementary material.

**Supplemental Figure S1:** Integrated analysis of regression relationships among tested ceramides. Scatter plots illustrating the regression analyses between different ceramide species. Each plot features a regression line in red with its corresponding equation, showcasing the relationships between the ceramide pairs; CerC24:1 vs. CerC16:0, R^2^ = 0.18, *P* < 0.01 (**A**), CerC24:1 vs. CerC18:0, R^2^ = 0.14, *P* < 0.01 (**B**), CerC16:0 vs. CerC18:0 (**C**), R^2^ = 0.39, *P* < 0.01, and CerC24:0 vs. CerC22:0 (**D**), R2 = 0.5, *P* < 0.01.

## Data Availability

The datasets generated and/or analyzed during the current study are available from the corresponding authors upon request.
